# What’s the Big Deal? Responder Experiences of Large Animal Rescue in Australia 

**DOI:** 10.1371/currents.dis.71d34082943fa239dbfbf9597232c8a5

**Published:** 2015-01-22

**Authors:** Bradley Smith, Kirrilly Thompson, Melanie Taylor

**Affiliations:** School of Human Health and Social Sciences,Appleton Institute, Central Queensland University, Adelaide, South Australia, Australia; School of Human Health and Social Sciences,Appleton Institute, Central Queensland University, Adelaide, South Australia, Australia; Centre for Health Research, University of Western Sydney, Sydney, New South Wales, Australia

## Abstract

Background: The management of large animals during disasters and emergencies creates difficult operational environments for responders. The aims of this study were to identify the exact challenges faced by Australian emergency response personnel in their interactions with large animals and their owners, and to determine the readiness for large animal rescue (LAR) in Australia. Methods: A survey tool collected the views and experiences of a broad cross section of emergency services personnel operating across Australia and across all hazards. Data were collected from 156 responders including Australian emergency services personnel, emergency managers such as federal agricultural departments, and local government. Results: Overall, many of the respondents had serious concerns, and felt that there were significant issues in relation to LAR in Australia. These included the coordination of emergency care for animals, physical management of large animals, inter-agency coordination, and dealing with animal owners. Very few respondents had received any formal training in LAR, with an overwhelming majority indicating they would attend formal training if it were made available. Discussion: Results help to guide the development of evidence-informed support tools to assist operational response and community engagement, and the production of professional development resources.

## Introduction

In industrialised countries, most large animals are kept in peri-urban or rural areas. During natural disasters such as wild fire and flood, large domestic animals (e.g., cattle, sheep, pigs) and wildlife (e.g., kangaroos, emu, camels) can find themselves in all manner of common and novel entrapment situations from which they require rescue. Common scenarios include falling into a trench or water, becoming entrapped in mud, entangled in fencing, or injured and unable to move. Large animals may also obstruct emergency responders in their duty, for example, animals released from properties can obstruct roadways or lead to vehicle accidents. Situations requiring large animal rescue (LAR) are not just restricted to rural areas as large animals also frequent cities, as police mounts or carriage horses^1^ or on inner city race courses where they frequently share spaces with road users and pedestrians who have little experience handling large animals. Other large domestic animals are transported through or around cities. Sometimes they experience traffic collisions or rollovers. The number of animals and/or incidents that can occur at the same time, and the scale of the operation to assist can be overwhelming for emergency responders. For these reasons, LAR is relevant to all emergency responders, regardless of their geographical base.

The management of large animals during disasters presents several challenges to emergency services, and presents dangerous situations for both the animals and the responders. The size, strength, unpredictability and diverse behavioural responses of large animals – even within the same species (i.e., from wild to tame domestic animals), and in response to specific stressors (i.e., entrapment, the rescue environment, medications) - pose serious threats to the safety of owners, responders and the general public. Successful LAR calls for multidisciplinary team efforts with clearly defined roles where different professionals such as veterinarians and emergency responders rely on one another’s skills and experience to keep themselves and each other safe. For example, emergency services need to ensure the safe access of a veterinarian to a trapped animal to administer sedation or medication, but a veterinarian needs to make sure that the dosage does not make the animal dangerous to responders, or that he/she is not put in a situation from which they may also need rescue. Successful rescue requires emergency service personnel with specialist training, access to resources, local knowledge, management of animal owners, and coordination among various emergency service providers.

Formalised LAR training programmes and Rescue Teams have been developed and refined internationally. Organisations include The British Animal Rescue and Trauma Care Association and various Technical Large Animal Rescue Teams and Trainers in North America; resulting in technical support and specialist rescue and training equipment such as strops, glides and mannequins. Their work is informed by several well-respected manuals on the topic^2^ ^3^. Much of the academic literature on LAR focuses on technological interventions^4^ ^5^, approaches^6^, skills and training^7^. The contexts range from emergencies such as livestock transport accidents^8^, animal-vehicle accidents^9^ ^10^ and livestock rescue^11^.

However, we know much less about the human component of LAR, particularly from the perspective of those emergency personnel that are often responsible and accountable, and especially in an Australian context.****To determine current attitudes towards, and experience with LAR in Australia, we conducted a survey of Australian responders and Emergency services personnel. In the absence of any previous research in Australia, we were particularly interested in 1) problems or difficulties around the management of animals in disasters in general, 2) previous experience with LAR, 3) previous experience with LAR training, interest in (further) LAR training and 4) any issues that were not anticipated from the literature.

## Materials and Methods

A questionnaire was designed to capture the experiences and attitudes of a broad range of responder groups including, but not limited to, emergency services personnel (see^14^ for further details). The data presented here are part of a larger study aimed at gathering information from a broad cross-section of responders across Australia and across all hazards and in relation to the broad topic of animal management during disasters.

The study did not undertake a statistically representative survey. Rather, it was intended to be exploratory in nature, in order to collect a broad range of information from emergency responders in relation to the management of animals and animal owners. Both opportunistic and targeted snowballing approaches were taken to recruit participants. A paper version of the questionnaire was used to gather responses from personnel attending the Australian Community Engagement and Fire Awareness Conference in May 2014, and later a small number of Emergency Media professionals were recruited at the Emergency Media and Public Affairs Conference in early June 2014. Following this, a link to an online version of the questionnaire was sent to a number of emergency service contacts with an email and a request to approach members of their organisations to invite them to take part in the survey. No request for follow-up or reminders were issued.

A mixed methods approach was taken, with both open and closed-ended responses included in the survey. Generic questions were asked in order to remain relevant to a wide range of personnel operating across all Australian States and Territories and all hazards. The survey was designed to cover the attitudes of their organisation’s role and responsibility in relation to animal management, their view of the problems and challenges encountered by themselves or their organisation, and their experiences with animal owners. Four specific questions were included relating to LAR and their level of experience, training, and capability (see results). To gain more detailed or unanticipated information, participants were also asked: “Do you have any further comments on large animal rescue?” None of the questions were mandatory, enabling respondents to provide information freely.

A total of 156 emergency responders participated in this survey (48 paper based and 108 online). Most respondents (74.4%) were from emergency services agencies, with the remainder from other ‘types’ of responders or emergency managers such as local government (council) officers (9%), Federal or State Government (primary industries) (3.2%), and animal organisations (2.6%). The majority of the emergency services sample comprised personnel from five agencies: New South Wales Rural Fire Service (30.8%), Queensland Fire and Emergency Services (18.6%), Department of Fire and Emergency Services (Western Australia) (15.4%); Country Fire Authority (Victoria)(5.8%); New South Wales State Emergency Service (2.6%). All states and territories were represented, with concentrations from New South Wales (32.7%), Queensland (30.7%), and Western Australia (22.2%).

Over half of the respondents were volunteers (66.7%), a quarter (26.7%) were salaried personnel, and a few (6.7%) indicated that they had another type of employment status. On further investigation this latter group comprised individuals who were typically members of more than one organisation, e.g. ‘*salaried DFES and volunteer SES’*. Overall, the sample comprised experienced emergency service personnel, with half (48.0%) having served in their organisation for 11 or more years (29.6% for 11-20 years, and 18.4% for more than 20 years). A quarter (24.4%) had served for five years or less.

The gender of the sample was fairly evenly distributed, with 53.9% male and 45.4% female. Half the sample (52.0%) was aged over 50, one quarter (24.3%) aged 41-50, and the remaining quarter (23%) aged 40 or under. Around three quarters owned household pets (71.2%) and 38.5% owned outdoor animals/pets (e.g. larger animals such as horses, cattle, and/or smaller animals such as chickens).

Only 30.5 per cent of respondents were aware of formal animal emergency response arrangements in their state emergency management plan, a further 20.8 per cent remained unsure, and almost half (48.7%) were not aware of these arrangements.

## Results

**Experience and confidence with large animal rescue**

Participants were first asked, "Do you have any experience with large animal rescue". In response, a total of 34.6 per cent reported that they had experience, with 52.6 per cent reported having no experience, and 12.8% indicating the question was *‘not applicable*’ to them. Non-applicable responses were retained in the subsequent analysis because they still remained indirectly involved in LAR. For example, one participant recognised that a lack of previous experience did not negate the possibility of future issues with LAR:

"As our brigade is near xxx Rd, it is probable that at some stage we will have an MVA [motor vehicle accident] including animal transport, or horses/cattle/alpaca left to fend for themselves during a fire event - gates open".

Another participant was more staunch in his/her support of LAR as an issue:

"This is a very important issue and it is way past time for us as emergency responders to spend more time researching it".

In this study, animals mentioned in relation to LAR included horses, livestock (sheep, cattle, pigs), and kangaroos.

Emergency responders were asked to rate “How capable would you feel dealing with a LAR e.g., cattle truck crash, trapped horse?” using a 5-point Likert scale including 1 = ‘*not at all capable’*, 2 = ‘*somewhat’*, 3 = ‘*moderately’*, 4 = *‘very’*, and 5 = ‘*extremely capable’*. Most respondents believed they were ‘*moderately capable*’ (27.1%), followed closely by ‘*not at all capable’* (25.8%), ‘*somewhat capable’ *(24.5%), very capable (17.4%), and only 5.2% ‘*extremely capable*’. The greater the mean score, the more capable the respondents felt. Responses ranged from 1 to 5, with the mean score 2.53 (±1.197).

In order to determine factors that may lead to respondents feeling capable when dealing with LAR, a series of Independent-samples Mann-Whitney U tests was conducted. Respondents that had experience with LAR *U* = 791.000, *p* < 0.001, *r* = -0.55; had received LAR training *U* = 433.500, *p* < 0.001, *r* = -0.32; and who had owned an outside pet (e.g., horses) *U* = 3920.000, *p* < 0.001, *r* = 0.34, reported significantly higher scores on their ability (capability) to deal with LARs. Effect sizes were moderate to large.

Emergency responders that were experienced with large animals, had higher self confidence in terms of dealing with LARs. This was demonstrated very clearly in the following free text responses:

"Having grown up on the land I have no problem assisting with what ever needs to be done to 'manage' the livestock".

"Most rural fire fighters have a fair understanding of the way stock animals react and behave".

"As a horse owner I would have no problems".

**Large animal rescue training**

Respondents were asked several questions about formal training. Only 10.4 per cent reported having received any formal training (“Have you received any LAR training?”); with 81.8 per cent having received none, and 7.8 per cent responded ‘*not applicable’*. However, respondents reported an overwhelming desire to attend training if given the opportunity. When asked “If your organization offered a large animal rescue course would you attend it?”, 67.1 per cent answered ‘*yes’*, 19.1 per cent answered ‘*no’*, and 13.8 per cent ‘*not applicable’*. Furthermore, all respondents that had received LAR training indicated they would attend further training (15/15). Of those that hadn’t received any training, 75.0% indicated they would attend training if given the opportunity.

Whilst respondents were not directly asked what kind of training they required, they commented on training needs throughout the survey. This included the provision of exemplar case studies of well laid plans for animals, awareness and risk training for emergency services, up-skilling crews on LAR techniques (e.g. handling and safety), dealing with injured animals, awareness of local resources (such as cranes, trucking companies, agriculture food suppliers), and establishing better relationships with animal organisations. Participants’ responses to free text questions throughout the survey provided strong evidence for four particular areas that training should address: animal handling, multi-agency coordination, interactions with animal owners and understanding that not all animal owners are the same.

***1. Training needs to cover animal handling skills***

Without formal LAR training requirements in Australia, many large animal rescues appeared to rely on an element of luck.

"Our own … crews are not all aware and or able to deal with animals. There is no training and it is just lucky if you have a farmer /horse person or something on your truck".

In recognition of the unreliability of animal handling expertise within a given crew, and the inherent desire of some to save animals, many respondents called for more animal specific training:

"It is essential training for emergency responders even if just to keep the responders safe. People will always try and help animals and we need to train people to do it safely". 

The importance of training to build confidence in animal handling is not only for the safety of responders. It might be the factor that gives animals their first chance at survival:

"Many animals are shot because it’s all too hard to face the rescue process, most vets are not experienced or trained in these matters and quite often the emotions involved for the owners are massive and therefore they can’t offer much in terms of constructive advice". 

This comment highlights exactly what is at stake in a situation requiring multiple stakeholders to collaborate effectively. Euthanizing animals is not only unnecessary with adequate LAR training, it is catastrophic for public relations – especially in an era of social networking and immediate media coverage.

***2. Training needs to facilitate effective interactions between multiple agencies***

Despite being prompted to discuss challenges with inter-agency coordination, respondents recognised that LAR benefitted from the collaboration of expert agencies:

"Large Animal Rescue involves a myriad of considerations (from responder and public safety, to issues of animal welfare and public perception) that either doesn’t exist or are less significant with smaller animal incidents. Thus, having trained responders either on site or available for consultation is vital. This really should be a priority for all Fire and Rescue departments". 

One respondent acknowledged not only the need for interagency collaboration, but a means to ensure that collaboration is effective:

"In the instance of cattle truck crash, trapped horses, bushfire through large property where there are livestock etc., it is important that government departments work with local rescue departments instead of hindering them. It is important that local government realise that there is a need, and make appropriate contact to reach out to transport companies, vets and the like so that in a situation like that, animals can be transported on mass [sic] out of the emergency zone. It would be very handy if during a situation like that, emergency groups … could just commandeer livestock transportation trucks, or similar". 

Transporting injured animals was a concern for several respondents, with recognised implications for further incidents:

"After accidents animals left at the scene as they cannot be taken in the ambulance and fire truck not fitted to carry distressed animals". 

To provide clarity in the potential chaos of an emergency, the training of emergency personnel needs to include an understanding of the identified roles, responsibilities and functions of multiple agencies is required.

"Training in these scenarios - sheep trucks, horse floats, animal management and chain of custody of animal in an accident as fire trucks, police and ambulances are not equipped to transport these so who to call, etc".

***3. Training needs to facilitate effective interactions with animal owners***

When asked for any specific problems or challenges with animal owners, several participants noted the ways in which large animal rescues could quickly become ‘large animal owner’ rescues, because of the reluctance of owners to hand-over responsibility of their animal:

"Animal owners, particularly horse owners, are reluctant, at times, to remove themselves from danger and hand over the control of a rescue to our trained responders. Often their lack of appropriate PPE and their emotional, irrational response to their distressed animal is an added challenge during rescues potentially putting the owner and others involved at greater risk. Often when we arrive on scene the owner is part way through attempting a rescue, sometimes in a very contradictory way to the way we are trained to deal with the task". 

One participant commented on an inherent tension in prioritising human life over animals, when animals could be so important to human lives:

"As a fire service our priority is people's life and obviously many people have significant bonds to animals, which cause A LOT of stress and angst during incidents like fire and floods". 

The implications for human health and safety of not being assisted or enabled to rescue and relocate their animals was recognised in the following comments:

"Owners refusing medical treatment because they are worried about their animal being left on the side of the road or how they will get them back (when airlifted)".

"Animal owners cannot present to the Welfare Centre as the Centre is not set up to take animals. People then refuse to evacuate from their homes as they refuse to leave their animals". 

These difficulties were considered largely preventable:

"Bush fire response - owners attempting to enter dangerous evacuated areas to tend animals/check on welfare, especially, where these could have been evacuated prior to impact".

However, many responders lamented the fact that large animal owners had no – or insufficient – emergency plans:

"Most large animal owners do not have an emergency plan that can be adopted especially for night time events".

Further complicating participant experience that owners are reluctant to hand over responsibility of their animals during an emergency rescue, some attributed a lack of natural disaster and emergency planning to owner confusion over responsibility for large animals during a disaster or emergency, even though owners in Australia are legally responsible:

"The owners think it is the volunteer’s responsibility to move animals and also find a place to put them in times of disaster".

Despite knowing that responsibility for animals rests with the owners, they tend to consider and protect large animals when possible, potentially reinforcing the owner beliefs with which they are burdened:

"To my mind it would be the owner’s responsibility to make sure their animals are safely removed. Not sure what training would assist us and the time involved, having said that we would assist in whatever way we could anyway".

However, there was evidence that effective owner-responder relations can be developed and maintained to facilitate successful large animal rescue:

"When responding to calls for assistance for large animal rescues recently we have had excellent rapport with the animal owners. They’ve been willing to assist both physically and financially in the rescue operation e.g. hiring a crane etc. We have had issues in the past during evacuations where animal owners were ill prepared, and/or wouldn't leave their homes or properties without the animal. More often we have problems with animal activist groups or vets. We have now identified a need to work with large animal owners on developing clear and explicit preparedness plans for flood emergencies". 

***4. Training needs to recognise that not all large animal owners are the same: the case of horse owners***

As noted in an earlier quotation regarding owner reluctance to ‘stand back’ from the rescue of their animals, horse owners were singled out by some emergency responders as a particularly problematic group. This was attributed to their strong attachment towards their horses, and being highly emotional.

"Horsey people are a hard group to deal with. They are totally focused on their horses. Most have none or very little bush fire preparation for their animals. They do not seem to plan 'when' and where they will take their horses. 

One particular issue with horse owners referred to a long held belief amongst the horse community that external property gates should be left open for horses to flee during fires. This advice is based on an implicit understanding that horses will ‘naturally’ know the ‘best’ place to go. However this can cause various problems for people and horses.

"The main point is being able to contact the owners during the emergency to facilitate movement / transfer. Some animals can be let out through the gate e.g. cattle, however it is different with horses". 

"In this rural area the general consensus is that to turn the animals loose and worry about catching them later. However this creates dangerous situations on the roads with cattle and horses bolting from the disaster area". 

This behaviour of releasing horses during disasters can very quickly lead to the need for large animal rescues where animals become trapped, for example in fencing obscured by floodwaters or smoke. Fires can have the effect of loosening horses simply by razing fences.

Human-horse relationships are distinctive amongst human-animal relationships for many reasons^12^. One of the relevant differences in relation to large animal rescue and emergency management, and in comparison with livestock or pets, is the increased percentage of horse owners not keeping their animals at their primary residence. Many horses are kept in livery, known in Australia as agistment properties. Horse agistment presents two main challenges for emergency management. First, owners may travel from more to less urbanised areas to rescue their horses:

"I have experienced the following: Roads used for evacuating communities blocked by horse floats responding to assist an unprepared stud owner, people helping to evacuate friends horses with no plan or idea of the area and not knowing the roads into or out of the area they have gone into to assist. Non emergency personnel removing horses/stock from properties without the permission of the owner with no notice of where they have taken the animals and how they can be recovered". 

Second, owners from non-agricultural backgrounds may lack basic natural disaster or animal emergency awareness:

"I have horses on agistment and other animals at home, as well as being a member with [two response agencies], I spend a lot of time trying to get horse owners to think about their horses in the event of a bushfire, most live in the suburbs and have absolutely no idea of the time line or risks involved in attending to horses in bushfires nor are many of them able to deal with the emotions involved in planning for their best friend (who others would just call a horse) in the event of a fire. Some refuse to think about it and others over react". 

These issues were borne out in the 2003 Canberra bushfires, and led to educational resources targeting agisters and agistees^13^.

Whilst our study identified four key areas for LAR training to address, finding time to conduct training might be difficult, particularly for agencies that are not specifically focused on animal rescue:

*"Unfortunately, as most fire fighters in my State are volunteer - we don't have the time to complete training that isn't essential to fighting fires".* ****

## Discussion

This study provides the first look into the emergency management of large animals in Australia, from the perspectives of emergency responders. It aimed to determine current attitudes towards, and experience with LAR in Australia. In particular, there was a need to understand 1) problems or difficulties around the management of animals in disasters in general, 2) previous experience with LAR, 3) previous involvement in LAR training, interest in (further) LAR training and 4) any other important issues that were not anticipated from the literature.

In general, many of the respondents had serious concerns, and felt that there were significant issues in relation to LAR in Australia. These included in the coordination of emergency care for animals, physical management of large animals, inter-agency coordination, and dealing with animal owners.

Around one-third of the responders had direct experience with LAR, however most only rated their ability level to deal with large animals as ‘*moderate*’. Only a handful rated their ability level as ‘*extremely capable*’. The prevalence of LARs is difficult to gauge, particularly because large and small animals are often not distinguished in incident reports. Therefore, it is difficult to determine whether the level of experience expressed by this sample is reflective of the number of agencies and staff required to meet the number of actual incident. It also makes the evaluation of training impossible, especially on injury data related to LAR. Regardless, these incidents are complex, and occur on a regular basis, so coordinated and evidence based approaches need to be facilitated and supported.

Experience of owning large animals was related to self-rated levels of capability in dealing with large animals in emergency situations. Although experience with large animals is likely to assist general animal handling, few animal owners have specialist training in handling stressed animals in challenging situations. Thus, high self-efficacy in such scenarios may place some responders in increased risk of physical danger. Trainers and educators should be aware that those with prior large animal handling experience may: have a false sense of competency; be complacent about personal safety around large animals; assume that the skills required for one type of large animal are relevant to others; take more risks during LAR; and not consider themselves in need of training.

Even though LARs were not a core component of the roles of many of the respondents or response organisations, respondents stressed the lack of specialised training in how to deal with such incidents. Very few had received any formal training at all, with an overwhelming majority indicating they would attend formal training if it were made available. This included personnel that had previously attended training. Training in this case, refers to hands-on scenario based training simulations, the use of specialist equipment, knowledge of legislation and of local organisations, and familiarity with local resources that might be able to be used (e.g., local crane operator, and truck drivers). In addition to training, a number of strategies were identified that may assist in the improvement of LAR in Australia: increased number of resources (special equipment, trucks, cranes); level of cooperation between agencies; education of the public; and allowing animals in evacuation centres. However, adding to the training demands of a workforce comprising mostly of volunteers is likely to be difficult. Therefore, education and training initiatives should also be directed towards large animal owners.

Animal owners were depicted by emergency responders as being either a help or a hindrance, particularly when they were not well prepared or didn’t adhere to advice. This study suggests that emergency personnel would benefit from training and support in how to manage animal owners, particularly when the owners are experiencing a wide range of emotions such as distress, fear and anger. Horse owners were singled out as particularly challenging due to their highly emotional animal bond, but also because horses can be difficult to evacuate and can cause serious risk if released. Despite the difficulties associated with horses on agistment properties, the concentrated network of horse owners in one location could be put to use in group behaviour change, role modelling and information dissemination. Further, incentives could be provided to, or accrued by, agisters or agistees that have a LAR training or an emergency action plan.

LAR scenarios are complex and extremely diverse. Thus, it is important that we define the parameters of LAR, because there are other ‘large’ scale animal emergencies, that can occur in both regional and urban areas. Also, what animals come under the banner of LAR may need to be fleshed out. Chickens, for example, are not large, but a scenario of a truck rollover of one thousand could benefit greatly from LAR skills and multiagency collaboration.

**Limitations and future research**

The sample used in this study was self-selected using opportunistic and targeted sampling techniques. As such, the data presented here do not represent the position of personnel across Australian emergency response organisations, but is merely indicative of some of them. The intention of this study was to provide an exploratory and preliminary look at issues and concerns surrounding large animal rescue as reported by the industry themselves. It is likely that those who took part in the survey were either more interested or engaged in this area or felt they had a grievance to express or contribution to make. Further studies need to ensure coverage of all emergency services or other organisations involved in the management of LARs to determine whether the same challenges identified here are universal. Further analysis is also needed in terms of coordination between responder organisations, and the effectiveness of training programs for emergency response personnel. We did not provide a definition of LAR and it is uncertain whether definitions of LAR are unanimous across different response organisations. Results from this, and future studies can help to guide the development of evidence-informed support tools to assist operational response and community engagement, and the production of professional development resources. Lastly, we recommend similar studies, but with other agencies and organisations that are involved/coordinate with government and emergency service organisations such as veterinarians, and also community (e.g. cattle farmers and especially horse owners).

## Conclusion

LAR is clearly a ‘big deal’ to animal owners, emergency response personnel. The management of large animals during disasters creates difficult operational environments that present several challenges to emergency responders. In order to be successful, it requires emergency responder personnel that have specialist training, access to resources, local knowledge, and coordination among various emergency service providers. This study would seem to suggest that the way forwards is not to ask emergency responders to change their current model or compromise their mandate to privilege human life, but rather to coordinate more effectively with organisations and services that are already established and experienced dealing with large animals in natural disasters, and also to build working relationships with owners. Unfortunately, the cost of not taking action in terms of coordinated LAR, is poor outcomes for animal welfare, the community, and emergency responders themselves.
